# Economic Stress at Work: Its Impact over Absenteeism and Innovation

**DOI:** 10.3390/ijerph18105265

**Published:** 2021-05-15

**Authors:** Martin Sanchez-Gomez, Gabriele Giorgi, Georgia Libera Finstad, Federico Alessio, Antonio Ariza-Montes, Giulio Arcangeli, Nicola Mucci

**Affiliations:** 1 Department of Evolutionary, Educational, Social Psychology and Methodology, Universitat Jaume I, 12071 Castellón de la Plana, Spain; sanchgom@uji.es; 2 Department of Human Sciences, European University of Rome, Via degli Aldobrandeschi, 190, 00163 Rome, Italy; gabriele.giorgi@unier.it; 3 Business@Health Laboratory, European University of Rome, Via degli Aldobrandeschi, 190, 00163 Rome, Italy; g.liberafinstad@gmail.com (G.L.F.); federico.alessio94@gmail.com (F.A.); 4 Management Department, Universidad Loyola Andalucía, 14004 Cordoba, Spain; ariza@uloyola.es; 5 Department of Business Administration, Universidad Autónoma de Chile, Santiago 7500912, Chile; 6 Department of Experimental and Clinical Medicine, University of Florence, Largo Piero Palagi 1, 50139 Florence, Italy; giulio.arcangeli@unifi.it

**Keywords:** economic stress, work related stress, absenteeism, innovation, innovative behavior, mental health

## Abstract

Economic stress has been recognized as a major threat to the well-being and performance of workers, especially during times of global economic crisis. An interesting and relatively unexplored research topic concerns the associations between economic stress and employee job outcomes such as innovative behaviors, indispensable for business survival. The aim of the present study was to investigate the relationship between economic stress, absenteeism and innovation. We considered both a direct and a mediation hypothesis and hypothesized that economic stress can have a negative influence on innovation directly and indirectly through increased absenteeism. A cross-sectional study was performed during 2018 and 2019 in an Italian food factory. A sample of 578 employees completed the Stress Questionnaire, the Janssen’s nine-item scale and a single-item regarding absenteeism. All relationships are supported by empirical data. As expected, the results indicated that economic stress is negatively related to innovation and positively related to absenteeism, which, in turn, plays a mediating role in the relationship between economic stress and innovative behavior. Herewith, those employees with higher levels of economic stress show higher levels of absenteeism contributing at the same time to a decrease in innovative behaviors. These findings show the importance of economic stress in understanding individual work outcomes and highlight the need to promote adequate intervention programs.

## 1. Introduction

### 1.1. Economic Stress and Its Consequences

The psychological health and well-being of workers can be affected by several emerging and re-emerging occupational risks, especially during a financially unfavorable period of global crisis. The focus of organizational research has therefore shifted to take into account the effects of the macroeconomic environment. In detail, some researchers demonstrated that the economic crisis negatively affects workers’ psychological well-being through negative outcomes such as fear of non-employability, fear of the crisis, job insecurity and job loss [1,2]. In this regard, Probst and colleagues [3] (p. 268) defined economic stress as “aspects of economic life that are potential stressors that consist of both objective and subjective components”. Economic stress can have a major impact on workers’ health, and as early as 1967, Pierce’s [4] longitudinal study had discovered a relationship between changes in the economy for the years 1919 to 1940 and the suicide rate one year later. Indeed, economic changes impose necessary adaptive behaviors related to short-term emotional disequilibrium and stress, in turn, related to a higher incidence of medical and health problems [5,6,7,8]. The 2008 global economic crisis has renewed the attention of the academic literature on the relationship between economic stress and workers’ health [9,10,11,12]. For example, the systematic review conducted by Mucci et al. [13] found a clear correlation between workers’ mental health and the economic crisis. Factors such as increased unemployment, increased workload, staff reduction and wages reduction were associated with the onset of mood disorders, anxiety, depression, dysthymia and suicide, and profoundly affected the general health of workers. Workers employed in a company facing economic crisis or recession may perceive that crisis as a threat associated with potential losses, leading to psychological strain such as distress, anxiety and depression [13,14,15]. Ultimately, an interesting and relatively unexplored research topic concerns the associations between economic stress and employee job outcomes such as innovative behaviors, indispensable for business survival [16]. With regard to the Italian context, according to the economic surveys of the Organization for Economic Co-operation and Development (OECD) of 2017 and 2019, the economic and social fabric is still affected by the aftermath of the 2008 crisis. Since 2008, real gross domestic product (GDP) per capita has fallen by 10% and still remains lower than the pre-crisis period’s one, overall displaying little variation in two decades. Productivity continues to show a negative trend, investments are at only 80% of the pre-crisis average and the unemployment rate is one of the lowest by international standards while the Italian public debt/GDP ratio is the third-highest among the OECD countries [17,18]. This issue becomes even more salient in this period of global economic crisis caused by the recent Coronavirus Disease 2019 (COVID-19) pandemic. Work-related factors, such as being employed in front-line activities, working in the health sector or belonging to a fragile population (migrant workers, older workers) seem to profoundly affect workers’ outcomes, especially when acting together with personal and environmental factors such as being previously affected by psychological disorders, living in overpopulated areas, being subjected to isolation measures and lockdown policies [19]. The International Labor Office (ILO) has recommended that factors such as fear of job loss, pay cuts, layoffs and reduced benefits shall be properly assessed [20]. Despite these recommendations, the assessment of economic-related factors in the workplace lacks reliable methods and has often been performed by outdated tools [21]. In this regard, Giorgi and colleagues [22] proposed an innovative tool aimed at measuring fear of economic crisis defined as “the individual’s perception of the crisis’ potential negative effect on his or her job, as well as on his or her organization” (p. 139). 

### 1.2. Absenteeism and Withdrawal Behaviors 

The findings described above show how the economic crisis can lead to detrimental consequences for employees. In this perspective, absenteeism is configured as one of the possible coping strategies associated with these circumstances [23,24]. Indeed, as early as 1955, Hill and Trist [25] had suggested that strain leads employees to withdraw with absences. According to the restorative model of absence, this coping strategy would allow employees to recover resources [26,27]. Absenteeism is part, together with lateness and turnover of the so-called “withdrawal behaviors,” which are defined as “physical removal from a particular workplace, either for part of a day, an entire day, or permanently” [28] (p. 233). Absences are a tedious problem both in material and immaterial terms, with costs up to 15% of the payroll [29] and negative consequences for work processes, productivity and job performance [30,31]. Absenteeism is typically studied at the individual level and its antecedents are commonly work-related such as increased workload, staff reduction, wages reduction, job insecurity [13], job expectations [32], level of pay, training, low satisfaction and low organizational commitment [33,34] task repetitiveness and amount of autonomy [35,36,37]. More recently, Montani and colleagues [24] proposed a cross-level framework in which psychological distress mediates the relationship between appraisal of economic crisis and work-unit absenteeism rate. Furthermore, it should be underscored that absenteeism can be classified into two types, in spite of this distinction not being unanimously accepted: involuntary (i.e., due to external circumstances such as medical/family issues) and voluntary (i.e., based on employee motivation) [24,33]. Some perspectives argue that there are different underlying mechanisms and that certain thresholds could help distinguish whether an absence is based on reasons beyond the employee’s control or conversely is voluntary. For instance, according to this perspective, longer absences (duration) are believed to be involuntary while short and repeated absences (frequency) may occur due to employee motivation. Nevertheless, other approaches consider these two aforementioned types of absenteeism as interrelated and do not support their division into clear-cut typologies, with repercussions for the measurement methods. This perspective does not believe in establishing a certain threshold or in using rigid criteria to distinguish between voluntary and involuntary absences, considering absenteeism as based on both individual and contextual factors. In addition, recent research showed that the two types of measurements (duration vs. frequency) have similar reliabilities, with an association between them close to unit adding the fact that meta-analytic studies have demonstrated that regardless of the type of absences measured, hindrance stressors have a positive association with absenteeism and withdrawal behaviors [24,27,33,38]. By virtue of this and following other authors [24], our measurement of absenteeism (methods section) does not distinguish between these two types, as it refers to the total number of sick leave days. This way of measuring absenteeism has been previously used and thus has proved its credentials as a valid and reliable methodology [39,40], emphasizing the point that it reduces the desirability bias [39,41].

### 1.3. Innovation at Work

Innovation is one of the fundamental variables for business success, especially in unpredictable and constantly evolving environments [16,42]. In order to obtain and maintain a competitive advantage, companies need to consider the entire cognitive capital at their disposal, stimulating the innovative work behaviors (IWB) of each employee [16,43]. Innovation can be defined as “a complex activity which proceeds from the conceptualization of a new idea to a solution of the problem and then to the actual utilization of economic or social value (…) innovation is not just the conception of a new idea, nor the invention of a new device, nor the development of a new idea, nor the development of a new market. The process is all of those things acting together in an integrated fashion” [44]. Taking this definition into account, workplace innovation can be understood as a broader process that includes the generation of ideas (creativity), their promotion and their realization within the work setting [42,45]. Indeed, innovation and creativity are cognitively demanding processes considered as extra-role behaviors [42]. To bring an innovative attitude at work, few things (social and individual antecedents) must be combined, such as employee behaviors and attitudes towards creativity [46] and perceived psychological safety about the organization [47]. In the process of innovation, individual characteristics such as a creative personality, openness to experience and high educational level, play a fundamental role. At the same time, the work environment represents a pivotal element in boosting innovation: innovative behavior is stimulated by contextual factors such as job complexity, job autonomy, time pressure, leadership and organizational climate for support and creativity [45]. On the other hand, some elements are capable of negatively influencing the innovation levels of employees, creating an unfavorable environment for the generation and implementation of new ideas. In this regard, individual innovation seems to be inhibited when people feel insecure and unsafe at work, although the mechanisms by which economic stress could affect workers’ innovation are not fully explained [48,49]. 

### 1.4. Economic Stress, Absenteeism and Innovation 

The purpose of the innovative framework of this study is to explore the relationships between economic stress, absenteeism and innovation, contributing to the existing literature. To devise our model, we carefully analyzed pre-existing theoretical frameworks and empirical results [16,24,43,50,51,52,53], although large amounts of data are not available due to the relative novelty of the topic. Because of this, we also relied on studies that include closely related constructs such as job insecurity, downsizing, threat of layoffs, creativity and productivity to investigate the relationships between the variables of interest, as already suggested by other authors [50,53,54]. Furthermore, we analyzed economic stress through the Stress Questionnaire developed by Giorgi and colleagues [55] whose items refer to both insecurity (“my professional future is uncertain because of the crisis”) and fear of downsizing (“I am scared that my organization, because of the economic crisis, is subjected to downsizing”).

As regards the relationship between economic stress and innovation, the available data suggest a generally negative relationship [16,43,53,56]. For example, the study of Bommer and Jalajas [57] highlighted a negative correlation between the threat of downsizing and the desire to provide innovative ideas to supervisors and to take risks. Similarly, Probst and colleagues [53], analyzed the effects of job insecurity on creativity in a simulated organizational setting (threat of lay-offs) and in a real setting (survey administered to 144 employees from 5 organizations). In both cases, the results showed a negative association between job insecurity and creative problem-solving skills. Moreover, Choi and colleagues [58] highlighted a negative relationship between job insecurity and innovation, also finding support for the role of organizational commitment and job satisfaction as partial mediators. Regarding the underlying mechanisms that can explain the relationship between economic stress and innovation, different models and hypotheses have been proposed. According to the early statements of Greenhalgh and Rosenblatt [59], employees living in a situation of job insecurity are less likely to solve tasks that go beyond their standard activities, according to what is called “disinvolvement syndrome”, which is clearly opposed to efforts needed for innovation [42]. Furthermore, in situations of economic stress, employees tend to adopt compliant behavior and avoid risky actions, leading to what is defined as risk-averse thinking, negatively associated with innovative efforts [60,61]. Following the Conservation of Resources Theory (COR), individuals can react to stress (in this case, economic stress) by using their resources to restore a favorable situation or by decreasing efforts (counterproductive condition for innovation) to conserve residual resources [26,62]. Other researchers state that stress leads to a reduction in the ability to process information and cognitive flexibility [53,62]. This is the perspective of the Threat-Rigidity Theory [63] according to which rigid forms of behavior are emitted when a threat is perceived. This theory is suggested as a partial explanation for the relationship between job insecurity and innovation by Niesen and colleagues [43]. According to their conceptualization, stress would lead to greater arousal and this in turn, following Zajonc’s [64] perspective, would lead to the expression of a dominant response. Hence, economic stress would lead, through arousal, to the emission of a dominant and inflexible response, inhibiting innovation. Therefore, based on previous research findings and theories, we hypothesize the existence of a negative relationship between economic stress and innovation. 

Furthermore, the perspective proposed by Niesen and colleagues [43] is adopted in the theoretical framework of Karatepe and colleagues [51] for the study of the relationship between economic stress and nonattendance behaviors (e.g., absenteeism). The authors, based on the threat-rigidity thesis [43] and COR theory [26], hypothesize that workers who feel insecure about their job may react to this threat by showing a habituated response in terms of greater absenteeism. Thus, economic stress would lead to a dominant response represented by an emotion-focused coping (as opposed to problem-focused coping according to the taxonomy of Lazarus and Folkman [65], also defined as non-strategic “hot” behavioral response according to Shoss and Probst [62]) such as withdrawal behaviors [43,51]. Hence, these behaviors could be used as a coping strategy as already pointed out in the case of the restorative model of absence. Indeed, the literature suggests a relationship between economic stress (e.g., job insecurity, downsizing) and withdrawal behaviors. For example, Sigursteinsdóttir and colleagues [66] studied the effects of the 2008 economic crisis on the sickness and sickness absence of 2356 workers and found that employees in downsized workplaces were 40% more likely to visit a doctor for work-related situations. The focus group interviews also highlighted how “part of the sickness absence was a way of coping with stressful situations” (p. 100). Similarly, another study [67] showed that a staff reduction of 1% was linked to an increase in sickness absence by an average of 9%. These results are confirmed by other authors who found that job insecurity is positively related to absenteeism [68,69] and negatively related to job performance [70]. 

On the other hand, absenteeism involves various detrimental organizational outcomes such as negative consequences on performance and productivity [30]. For example, some findings show that absenteeism is negatively associated with supervisors’ assessments of employee effort [71]. Data and research on the relationship between absenteeism and innovation are scarcer. However, an interesting recent research [52] analyzed the role of absenteeism and turnover as mediators in the relationship between diversity programs and performance and innovation. According to the hypotheses of the authors, absenteeism and turnover are associated with less effectiveness of workers and therefore, worse performance and less innovation. The results of the study support the theoretical model, showing that psychological and behavioral withdrawal (i.e., absenteeism) has detrimental effects on employee performance and innovation levels. 

These results and theoretical assumptions introduce our mediation hypothesis. Not only do we hypothesize a negative relationship between economic stress and innovation, but we hypothesize that economic stress can have a negative influence on innovation directly (rigid and inflexible habituated cognitive response combined with less effort) and indirectly (habituated behavioral response represented by withdrawal behaviors) through the mediation of absenteeism. In other words, the relationship between economic stress, absenteeism and innovation could be explained as follows: economic stress would lead employees to exhibit a dominant response (threat-rigidity theory) at the cognitive level preventing the flexibility necessary for innovation, and at the behavioral level with an emotion-focused coping such as absenteeism. At the same time, economic stress would lead employees to decrease cognitive efforts to save resources (COR theory), using absenteeism as a coping strategy for the same purpose. Absenteeism, in turn, would negatively affect employee innovation levels. 

### 1.5. The Present Study 

**Figure 1:**
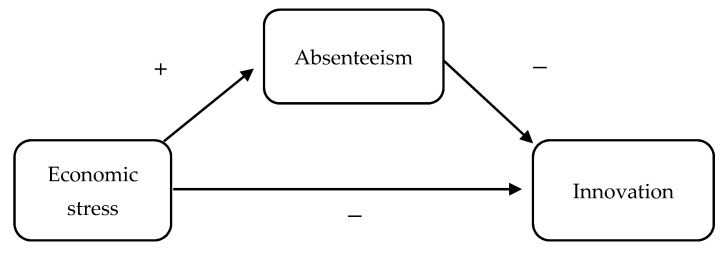
Mediation model proposed to test the associations between economic stress, absenteeism and innovation.

The main aim of the present study is to evaluate the relationship between economic stress and innovative work behaviors. Moreover, the mediating role of absenteeism was considered. Based on the threat-rigidity thesis [43], transactional theory of stress [65] COR theory [26] and the empirical results analyzed regarding economic stress, absenteeism and innovation, we hypothesize a negative relationship between economic stress and innovation and we hypothesize that employees with higher levels of economic stress will show higher levels of absenteeism, which in turn will contribute to decreased innovative behaviors. These hypotheses are shown in Figure 1. 

To the best of our knowledge, this is one of the first studies investigating the relationship between economic stress and innovation, as economic stressors (e.g., job insecurity) have long been ignored by employee innovation reviews [16]. Furthermore, this study analyzes the role of absenteeism as a possible mediator using an innovative framework.

## 2. Materials and Methods

### 2.1. Study Design and Population

Following a cross-sectional design, the study was carried out between 2018 and 2019 in an Italian food factory. A self-administered questionnaire was distributed to employees belonging to four operating locations, leading to a final sample of 578 subjects. A high percentage of the company participated in the research (between 80–85% according to the data provided by the company). The sample consists of employees with stable jobs and salaries; however, the organization also employs a certain proportion of seasonal workers.

Table 1 shows the socio-demographic characteristics of the sample.

### 2.2. Measures

#### 2.2.1. Economic Stress

The Stress Questionnaire (SQ) was used to evaluate economic stress. This tool was developed by Giorgi et al. [55] in order to assess several psychosocial variables associated with work-related stress. The SQ is based on Karasek’s job demand-control model and the Health and Safety Executive’s Management Standards for work-related stress [36,37]. Taking into account the literature on work-related stress, the SQ was further developed, adding emerging factors and risks, such as fear of crisis and non-employability [55]. The “fear of the economic crisis” dimension is composed of 5 items (e.g., I believe that the crisis will not affect my stay in the company) and uses a Likert scale ranging from 1 (strongly disagree) to 5 (strongly agree). Higher mean scores reflect a more negative appraisal of the economic crisis. The Cronbach’s alpha coefficient obtained was 0.76.

#### 2.2.2. Innovative Work Behavior 

To evaluate the innovative work behavior, the Janssen’s scale was used [42]. This nine-item questionnaire includes a Likert scale ranging from 1 (never) to 5 (always) and evaluates the frequency with which employees are involved in the three components of innovation: the generation of ideas (e.g., techniques or tools) the promotion of ideas (e.g., seeking approval for innovative ideas) and the realization of ideas (e.g., evaluating the usefulness of innovative ideas). Nevertheless, numerous studies support the unidimensionality of the innovative work behavior construct [72]. The Cronbach’s alpha coefficient had a value of 0.95. 

#### 2.2.3. Absenteeism

Absences from work were measured through a single item (how many days were you absent from work due to illness or medical checks last year?). Responses were classified from 1 to 5 according to the days of absence (i.e., none; from 1 to 9; 10–24; 25–99; 100–365). People were asked to report only sickness-related absences that were medically certified, which allowed for a focus on a homogeneous category of absences, as opposed to the wide variety of reasons for absences—including vacation, training, parental leave, trade union activity, and personal reasons. Johns and Miraglia [40] showed that self-report measures of absences are as valid and reliable as objective data, and can be used confidently for correlational studies inasmuch as the individual tendency to underreport absences does not affect the ranking of employees.

#### 2.2.4. Control Variables

Additionally, several items were introduced to obtain socio-demographic data (i.e., gender, organizational tenure, operational location, work area and schedule that could be associated with the main study variables [39,73]. 

### 2.3. Procedure

The sample was recruited during 2018 and 2019, when the above-mentioned Italian company was contacted to participate in a cross-sectional survey. Following the American Psychological Association’s (APA) Ethical Principles of Psychologists and Code of Conduct, participants received an explanation aimed at clarifying the voluntary and confidential nature of their collaboration in this research. All of them gave their consent to participate in the study. The questionnaires were administered on site by human resources staff and researchers, taking into account the recommendations of Wheeler and colleagues [74] for collecting data in organizations. All questionnaires were administered in Italian and all participants were able to read and speak this language fluently. The whole process was conducted in accordance with the Declaration of Helsinki. Given the observational nature of the study, and in the absence of any involvement of therapeutic medication, no formal approval of the Institutional Review Board of the local Ethics Committee was required. 

### 2.4. Data Analysis

The statistics software IBM SPSS^®^ (v. 25, package for Windows, SPSS Inc., Chicago, IL, USA) was used to analyze the data. First, we performed descriptive statistics, including mean and standard deviation as indicators of central tendency and dispersion, respectively. Moreover, reliability analyses were performed for the study variables. After analyzing Person’s correlations among economic stress, absenteeism and innovative work behavior, we conducted a mediation analysis to test the hypotheses (absenteeism plays a mediating role between economic stress and innovation). For this purpose, the macro PROCESS 3.3 [75] was applied. Following a bootstrap method with 10,000 data samples that generates 95% bias-corrected confidence intervals, it was possible to examine conditional models to predict direct and indirect effects between the variables. A path is statistically significant if the associated 95% confidence interval (CI; bias corrected) does not include zero. The goodness of fit achieved by the model was assessed through different criteria such as the root mean square error of approximation (RMSEA); the goodness-of-fit (GFI) index; and the comparative fit (CFI) index. RMSEA values below 0.05, and GFI and CFI values over 0.9, indicate a good fit [76].

## 3. Results

### 3.1. Descriptive Analysis

Table 2 shows the correlations, means, standard deviations, and reliabilities concerning the study variables. 

As shown in Table 2, a statistically significant correlation was found between economic stress and absenteeism (*r* = 0.11) and a statistically significant correlation was found between economic stress and innovative behavior (*r* = −0.17). Similarly, absenteeism and innovation are negatively related (*r* = −0.12), as expected. The same results were found for the correlations between the three sub-dimensions of innovation (i.e., generation, promotion and realization of ideas) and the variables of interest. Moreover, the analyses revealed that the three branches of innovation are strongly correlated, as expected. Finally, the results show a good level of reliability with Cronbach’s alpha coefficients between 0.76 and 0.95. 

### 3.2. Mediation Analyses

**Figure 2:**
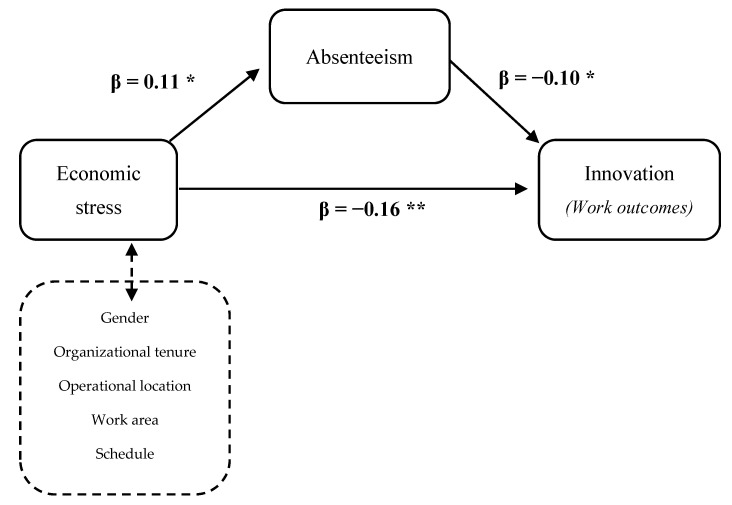
Mediation Model and relationship between variables. The path weights in the graph were standardized. ** *p* < 0.01. * *p* < 0.05.

A mediation analysis was performed to define the role of absenteeism. The standardized fit indices of the model showed a desirable fit (RMSEA = 0.045; GFI = 0.951; CFI = 0.90). Confidence intervals (CIs) were established using a multiple mediator model. Regarding the indirect effect, economic stress has a significant effect on absenteeism (β = 0.11; *p* < 0.02; 95% CI = 0.01, 0.11), which, in turn, shows a significant effect on innovation (β = −0.10; *p* ≤ 0.02; 95% CI = −1.85, −0.16). As can be seen in Figure 2, there is a significant direct effect of economic stress on innovative behavior (β = −0.16; *p* < 0.01: 95% CI = −0.14, −0.04). In conclusion, after controlling the effects of different socioeconomic variables, absenteeism partially mediated the relationship between economic stress and innovative behavior. The absenteeism variable can explain a 3% of the variance in innovative work behavior (R^2^ adj = 0.03; *p* < 0.01). 

## 4. Discussion 

In today’s highly unstable environment, employees face new psychosocial risks related to the pressures of the macroeconomic context. As a result, economic stress has been incorporated into work-related stress models as a key factor affecting employee well-being and several work outcomes. On the other side, the unfavorable economic environment is forcing organizations into growing competition. In this perspective, innovation becomes one of the crucial processes for business survival [1,15]. Economic stress has a major impact on workers’ mental health as factors such as fear of the crisis, fear of non-employability, staff reduction, wages reduction, job insecurity and job loss can have detrimental consequences [1,2,13]. Furthermore, economic stress as a hindrance stressor can trigger “hot” reactions in employees [62], and lead to withdrawal strategies [51]. At the same time, although economic stress is a highly destabilizing factor for job performance, this variable is generally overlooked in the innovation literature [16]. According to this, the main purpose of the present study was to investigate the relationship between economic stress, absenteeism and innovation. In particular, starting from previous research and theoretical models, we considered both a direct hypothesis and a mediation hypothesis, analyzing the mediating role of absenteeism. Drawing from the Threat-Rigidity thesis [43], COR theory [26], the transactional model of stress [65] and previous research on the topic [43,51,62] we hypothesized that economic stress can have a negative influence on innovation directly and indirectly through increased absenteeism. All relationships are supported by empirical data. The results of the mediation analyses show that there is a significant direct effect of economic stress on innovative behavior and that economic stress has a significant effect on absenteeism, which, in turn, shows a significant effect on innovation. Analyzing the direct relationship, the findings of this study support the emerging literature on the topic, underlining the central role of economic stress on employee innovation levels. As already described, there are several hypotheses and theories underlying the negative relationship between economic stress and innovation: compliant behavior and fear of risky actions, decreased cognitive efforts as a way to conserve resources in the face of a stressor and stereotyped responses and cognitive rigidity as a response to a threat. Regardless of the possible explanation, research on the topic suggest a negative pattern between economic stress and employees innovative work behaviors (as in the case of job insecurity, threat of lay-offs, downsizing) [16,53,56]. Indeed, economic stability is a pillar of employee well-being, essential for performance and innovation. Interestingly, albeit apparently counterintuitive, recent results show that some stressors (i.e., lack of job autonomy, job demands and role ambiguity) can have a positive and significant influence on the employees’ levels of innovativeness [77]. Hence, an interesting future direction could be to investigate whether this occurs in the case of economic stress, possibly establishing under what conditions economic stress becomes a type of stress capable of generating positive results for innovation [78]. In fact, the literature suggests the existence of a possible positive association between hindrance stressors and innovation, as these would function as a motivational engine leading to the implementation of more innovative behaviors [79]. For example, according to the person–environment fit theory engaging in innovative behaviors could improve employee adaptation in challenging environments [80] while the dual-tuning model states that both positive and negative emotions can lead to greater creativity [81].

Regarding the proposed mediation model, the results support the role of absenteeism as a mediator in the relationship between economic stress and innovative behavior. Multiple mediation analyses showed that economic stress has an indirect effect on innovation through absenteeism. It therefore appears that absenteeism may be a consequence of economic stress and may play a primary role in reducing innovation outcomes [47,48]. Thus, economically stressed professionals tend to increase the number of absences, which, in turn, reduces innovation outcomes. In line with COR theory [26] and Threat-rigidity theory [51], as well as previous studies, employees may react to economic stress even at the behavioral level with withdrawal behaviors, as a form of emotion-focused coping. Individuals can react to stress by decreasing efforts to conserve resources or through a stereotyped response following arousal, thus leading to a positive relationship between economic stress and nonattendance behaviors. As pointed out by previous research, absenteeism is associated with worse work outcomes in terms of performance [30,71]. In our case, we hypothesized a link between this withdrawal strategy and employee innovation levels, using an original framework [47,48,52]. Our results supported this hypothesis showing that absenteeism plays a key role in reducing innovative behaviors. Interestingly, research showed that a creative environment can, on the contrary, reduce the levels of absenteeism [41]. Future studies could further investigate the direction of the relationship between these variables. 

Based on these findings and previous research, it is plausible that increasing structural job resources will help employees develop innovative thoughts and nurture new ideas, products and processes, lowering absenteeism levels in a virtuous circle [51,82,83]. Companies should, as far as possible, ensure economic and social stability as a fundamental aspect of the strategy for continuous innovation. Regarding the novelty of this research and its contribution, to the best of our knowledge this is one of the first studies to explore the relationship between economic stress and innovation. Furthermore, we considered absenteeism as a possible mediator, including withdrawal strategies within our framework. Although many studies have investigated the influence of stress on work outcomes (e.g., performance), few of these have focused specifically on economic stress and its impact on innovation. Indeed, future research will have to analyze how the profound organizational changes that are modifying the world of work (e.g., new contractual and working methods) affect the complex relationship between the economic context and employees’ outcomes. 

### 4.1. Limitations and Future Research

The present study has some limitations which, however, could serve as starting points for future research. First of all, despite solid theoretical premises based on previous scientific findings, the cross-sectional study design does not allow for causal inferences about the relationships between the variables. Future longitudinal studies are needed to replicate these results and provide further insights about how economic stress influences innovation. Secondly, a possible limitation concerns the non-probabilistic sampling strategy used. Participants were recruited by the same company (with different locations) for convenience and this may have influenced the levels of generalizability. Participation was not mandatory, and this may have biased the sample as it could include the most active and cooperative workers, restricting the generalization of the results. However, our sample consisted of a heterogeneous group of individuals from a range of occupations. Future studies should further investigate the topic by analyzing different organizational realities. Indeed, for a broader understanding of how economic stress is associated with employee innovation, further studies should explore specific characteristics such as the sensitivity of the sector or company to the business cycle (e.g., the tourism and construction sectors are typically considered cyclical, while the public administration is less affected by the economic situation). In addition, the sample consisted only of Italian workers, limiting the generalizability to other populations. However, this way of obtaining data is usually used in organizational psychology and has shown good levels of validity and reliability [74]. Moreover, it should be noted that our sample includes employees with stable jobs and salaries. The findings of our study could therefore be different if replicated considering other types of workers (e.g., freelancers) who are more affected by the fluctuations of the economy. Lastly, even if in our sample 63% of participants work in the operational area, innovative work behaviors can also be observed in this type of occupational population [84]. In addition, economic stress may be a significant factor for operational workers, as some results suggest that economic rewards can improve IWB in blue-collar workers [85,86].

A further possible limitation of our model is not having taken into account other variables that can affect innovation at work. In fact, we did not check for the influence of individual factors related to innovation, such as creativity, the level of education and other variables related to the psychological profile of the subjects. Similarly, we did not check for the influence of contextual factors such as time pressure, the organizational climate for support or the level of conflict, which proved to be fundamental in understanding innovative behavior at work [45,46,47,87]. Furthermore, a possible limitation could lie in our measurement of absenteeism. Future studies could deepen the distinction between different types of absences with related measurement methods. Finally, a limitation that should be investigated through future research concerns slightly sub-threshold values obtained for some of the correlations. Despite these limitations, the results of this research support our theoretical hypotheses and provide interesting insights regarding the relationship between economic stress, innovation and absenteeism. 

### 4.2. Practical Implications 

The present study, beyond its limitations, offers important insights for organizational research and human resource management (HRM), broadening the knowledge of the consequences of economic stress. In order to avoid negative outcomes such as decreased well-being, poorer performance and lower levels of innovation, a regular assessment of work-related stress and psychosocial risks should be performed. After proper evaluation, the implementation of workplace health promotion programs is advisable. Indeed, these programs have demonstrated a positive return on investment [88]. Among these, programs aimed at developing emotional skills have shown a high impact on psychological distress and work performance [89,90]. This approach could help to enhance emotion management, reducing the influence that negative organizational and personal consequences—such as sick leave or constant rotations—have on the company’s competitiveness [91]. Furthermore, involving employees in decision-making processes could be useful to increase the sense of control and thus reduce the perception of job insecurity, with benefits also for creativity [92]. On the other hand, innovative behaviors can be stimulated through performance evaluation programs that reward the generation and implementation of new ideas and discourage withdrawal behaviors [51,58].

## 5. Conclusions

To sum up, this work investigates the relationship between economic stress, absenteeism and innovation. The results of the study conducted on a sample of Italian workers show that economic stress seems to be a negative predictor of innovation at work. Furthermore, economically stressed employees tend to be absent and this is negatively related to innovative work behaviors. These findings demonstrate the importance of economic stress in understanding individuals’ work outcomes and underline the need to provide support to employees affected by this type of stress. In this perspective, intervention programs should be implemented to reduce the impact of the economic crisis. This will help maintain and promote the health of workers, preventing the onset of mental disorders and helping them achieve the best possible performance. 

## Figures and Tables

**Table 1:** Individual characteristics of the sample.

Characteristics	
**Gender**	(%)
Men	62.3
Women	37.7
**Organizational Tenure**	(%)
5 years or less	15.4
6–15 years	24.7
16–25 years	22.1
More than 25 years	37.7
**Operational Location**	(%)
Company headquarters	40.5
Peripheral headquarters	59.5
**Area**	(%)
Office work	36.6
Operative	63.4
**Schedule**	(%)
Full day	40
Shift worker with night shift	49.8
Shift worker without night shift	10.2

Note: N = 578.

**Table 2:** Descriptive statistics and correlations between the study variables.

Variables	1	2	3	4	5	6
1. Economic stress						
2. Absenteeism	0.11 **					
3. Innovation	−0.17 **	−0.12 *				
4. Ideas generation	−0.14 **	−0.05	0.91 **			
5. Ideas promotion	−0.16 **	−0.10 **	0.93 **	0.80 **		
6. Ideas realization	−0.15 **	−0.09 **	0.93 **	0.79 **	0.84 **	
Mean	2.91	1.81	2.47	2.52	2.35	2.54
Standard Deviation	0.79	0.84	0.97	1.01	1.05	1.06
α	0.76		0.95	0.88	0.89	0.89

Note: N = 578. ** *p* < 0.01. * *p* < 0.05. α = Cronbach’s alpha.
